# Serial Changes in Knee Muscle Strength and Functional Performance After Anterior Cruciate Ligament Reconstruction: A Retrospective Cohort Study of 107 Patients

**DOI:** 10.3390/medicina62030489

**Published:** 2026-03-05

**Authors:** Seung Ik Cho, Ju Won Bae, Youngwook Sim, Dhong Won Lee, Byung Sun Park, Yu Bin Lee, Hun-Young Park, Eunjoo Lee, Sang Jin Yang, Joon Kyu Lee

**Affiliations:** 1 Department of Orthopaedic Surgery, Konkuk University Medical Center, 120-1, Neungdong-ro, Gwangjin-gu, Seoul 05030, Republic of Korea; bestjjo@hanmail.net (S.I.C.); bird200503@naver.com (J.W.B.); syossw@naver.com (Y.S.); wonbayo@naver.com (D.W.L.); qud516@naver.com (B.S.P.); 20240031@kuh.ac.kr (Y.B.L.); 2 Department of Sports Medicine and Science, Graduate School, Konkuk University, Seoul 05029, Republic of Korea; parkhy1980@konkuk.ac.kr (H.-Y.P.); eunjooo@konkuk.ac.kr (E.L.); 3 Physical Activity and Performance Institute, Konkuk University, Seoul 05029, Republic of Korea; 4 Department of Health and Exercise Management, Tongwon University, 26 Gyeongchung-daero, Gonjiam-eup, Gwangju-si 12813, Republic of Korea; sjyang@tw.ac.kr

**Keywords:** anterior cruciate ligament reconstruction, muscle strength, postural balance, Y-balance test, retrospective cohort study, longitudinal study

## Abstract

*Background and Objectives*: Anterior cruciate ligament (ACL) reconstruction (ACLR) is widely performed to restore knee stability and facilitate return to activity. However, recovery of muscle strength, balance, functional performance, and patient-reported outcomes does not occur uniformly over time. The longitudinal recovery trajectory across various functional areas during the first year after ACLR remains insufficiently characterized. *Materials and Methods*: We included 107 patients who underwent isolated unilateral ACLR using a hamstring autograft. Isokinetic knee extensor and flexor strength, postural stability, Y-Balance Test (YBT) performance, and subjective knee function scores were assessed post-injury (approximately six weeks after ACL injury and prior to ACLR) and at 3, 6, and 12 months postoperatively. All patients followed a standardized postoperative rehabilitation protocol. *Results*: Knee extensor strength deficit worsened at 3 months and remained present at 12 months. In contrast, knee flexor strength deficit decreased progressively and reached near-symmetrical values by 12 months. Sway path length decreased significantly over time in both limbs. In the operated limb, improvements plateaued after 6 months, and limb symmetry indices approached symmetry by 12 months. YBT limb symmetry indices demonstrated a non-linear recovery pattern. Anterior, posterolateral, and composite scores decreased at 3 months, recovered to post-injury levels by 6 months, and showed significant improvement at 12 months. Posteromedial reach did not decline at 3 months and improved significantly only at 12 months. Subjective knee function scores (Lysholm and IKDC) did not differ significantly between post-injury and 3-month assessments, but improved significantly from 6 months onward. Tegner activity scores gradually increased and returned to pre-injury levels by 12 months. *Conclusions*: Recovery after ACLR is prolonged and non-synchronous. Quadricep strength remains incompletely restored at 12 months, whereas hamstring strength recovers more favorably. Balance, functional performance, and subjective outcomes improve mainly after 6 months. These findings support the need for prolonged rehabilitation and serial, multidimensional functional assessments beyond time-based criteria.

## 1. Introduction

Anterior cruciate ligament (ACL) injury is one of the most common knee injuries among physically active adults participating in sports, and ACL reconstruction (ACLR) is widely accepted as the standard surgical treatment aimed at restoring functional stability and enabling return to sport (RTS) [1]. Despite successful restoration of mechanical stability, persistent deficits in muscle strength, functional asymmetry, and balance control remain clinically relevant issues for a prolonged period following ACLR [2,3]. Traditionally, decisions regarding RTS after ACLR have often been based primarily on postoperative time elapsed. Many previous studies have adopted time-based criteria, such as 6–9 months or 9–12 months after surgery, as key benchmarks for RTS decision-making [4]. However, increasing evidence suggests that time alone is insufficient to determine functional recovery, leading to a growing emphasis on objective functional assessments, including muscle strength testing and patient-reported outcome measures, in RTS decision-making [5]. Quadricep muscle strength recovery, in particular, is considered a critical determinant of functional outcome and RTS following ACLR. Numerous studies using isokinetic testing have demonstrated that deficits in knee extensor strength of the reconstructed limb persist at 6 months and even beyond 1 year postoperatively, and such asymmetries are closely associated with impaired performance during high-demand activities such as jumping, cutting, and landing [1,6]. While some studies have reported substantial recovery of quadricep strength by 12 months after surgery [2], others have found that clinically meaningful strength asymmetry remains at this time point, leaving ongoing debate regarding the true timeline of strength recovery [6]. In addition, longitudinal observations have shown that during the early postoperative period (approximately 3 months), Y-Balance Test (YBT) performance may improve despite a concurrent decline in quadricep strength compared with post-injury values [3]. Whether such early improvements represent meaningful functional recovery or simply transient adaptations remains unclear. At the intermediate stage of recovery, around 6 months after surgery, several studies have reported partial improvements in strength and functional asymmetry without complete normalization [6].

In addition to quadricep deficits, recovery of knee flexor (hamstring) strength is also clinically relevant after ACLR, particularly when hamstring tendons are harvested for grafting. A systematic review reported that although isokinetic hamstring strength may not differ between common graft configurations, deficits can be observed at deeper knee flexion angles (≥70°) during isometric testing, indicating that postoperative flexor strength outcomes may vary depending on graft harvest strategy and assessment protocol [7]. These findings suggest that flexor strength recovery may not parallel quadriceps recovery uniformly and that residual hamstring weakness could contribute to altered muscle balance and dynamic knee stability during functional tasks.

Beyond isolated muscle strength deficits, ACL injury and reconstruction may also disrupt sensorimotor control. The ACL contains mechanoreceptors that contribute to proprioceptive input, and injury to this structure can alter afferent signaling and neuromuscular coordination. A systematic review of postural control after ACLR reported evidence of impaired dynamic balance performance compared with healthy controls, particularly during more demanding tasks, although methodological heterogeneity was noted across studies [8]. Furthermore, a meta-analysis examining center-of-pressure outcomes during single-leg stance following ACL injury demonstrated increased sway magnitude and velocity compared with uninjured individuals, with substantial heterogeneity across studies [9]. In contrast, more recent evidence in ACLR populations suggests that certain single-leg stance measures may show only small or inconsistent differences compared with controls, highlighting that the magnitude of postural control deficits may depend on the specific assessment protocol and recovery stage [10].

Accordingly, dynamic balance assessments such as the YBT have gained attention as clinically feasible tools to evaluate integrated lower-limb function. A recent longitudinal study examined YBT limb symmetry in individuals after ACLR at 3 and 9 months postoperatively and explored its relationship with isokinetic strength and functional performance measures, supporting the relevance of YBT as a multidimensional indicator of recovery beyond isolated strength testing [11]. However, whether improvements in YBT performance reflect true restoration of neuromuscular control or compensatory strategies remains unclear.

More recently, interest has grown in long-term follow-up studies examining functional recovery trajectories beyond 1 year after ACLR. Using growth mixture modeling of IKDC scores measured preoperatively and at 1 and 2 years postoperatively, Gursoy et al. identified three distinct rate-of-recovery patterns, demonstrating that patients do not follow a uniform recovery trajectory and that functional improvement may vary substantially between individuals [12].

Taken together, muscle strength, balance ability, YBT performance, and patient-reported clinical outcomes all change over time following ACLR; however, the magnitude and timing of recovery vary depending on the assessment modality and follow-up interval [2]. In particular, uncertainty remains regarding the extent to which strength recovery and functional symmetry are restored at 1 year postoperatively [6]. Moreover, most existing studies have focused on cross-sectional analyses at specific time points, whereas longitudinal studies that comprehensively track strength, balance, YBT performance, and clinical outcomes within the same cohort over an extended period are limited. Despite the expanding literature on ACLR recovery, important gaps remain. Most studies have relied on isolated time point comparisons or have examined single outcome domains, limiting understanding of integrated recovery trajectories across early, mid, and late postoperative phases. Furthermore, the longitudinal interplay between muscle strength, balance, YBT performance, and patient-reported outcomes, including differences between composite and direction-specific measures, has not been fully clarified.

Therefore, the purpose of this study was to longitudinally evaluate active adults undergoing isolated ACLR over a 1-year follow-up period and to comprehensively analyze time-dependent changes in muscle strength, postural stability, YBT performance, and patient-reported clinical outcomes. By integrating various functional areas across repeated assessments, this study aimed to characterize the functional recovery trajectory following ACLR.

## 2. Materials and Methods

### 2.1. Study Design and Participants

This study was designed as a retrospective analysis of prospectively collected clinical assessment data obtained during routine clinical follow-up from the post-injury phase through 12 months postoperatively. This study was conducted and reported in accordance with the STROBE (Strengthening the Reporting of Observational Studies in Epidemiology) guidelines. A total of 164 consecutive patients who underwent primary ACLR at our institution between January 2019 and January 2024 were initially screened for eligibility. All patients underwent unilateral primary ACLR using a hamstring tendon autograft. Post-injury muscle strength and functional assessments were conducted approximately six weeks after ACL injury and prior to ACLR. To avoid misunderstanding, this time point is referred to as “post-injury” rather than “preoperative.” These assessments were repeated at 3, 6, and 12 months postoperatively using identical protocols. Only patients with isolated unilateral primary ACL injury were included, and cases with minimal partial meniscectomy that did not alter the standardized rehabilitation protocol were also included. Patients who underwent any concomitant procedures that could affect the standardized rehabilitation protocol were excluded. Patients were excluded if they met any of the following criteria: (1) ACLR with meniscus repair including ramp lesion; (2) history of prior ACLR on the opposite knee; (3) multi-ligament knee injury involving additional cruciate or collateral ligaments; (4) concomitant knee cartilage repair procedures; (5) postoperative complications that could affect rehabilitation outcomes (e.g., infection, graft failure, or reoperation); (6) neurological or musculoskeletal disorders affecting lower limb function; (7) inability to complete isokinetic strength testing, balance assessment, or functional performance tests; or (8) incomplete clinical or follow-up data. These exclusion criteria were established based on commonly applied standards in prior ACLR outcome studies to minimize confounding factors related to neuromuscular control, functional recovery, and RTS assessment.

**Figure 1:**
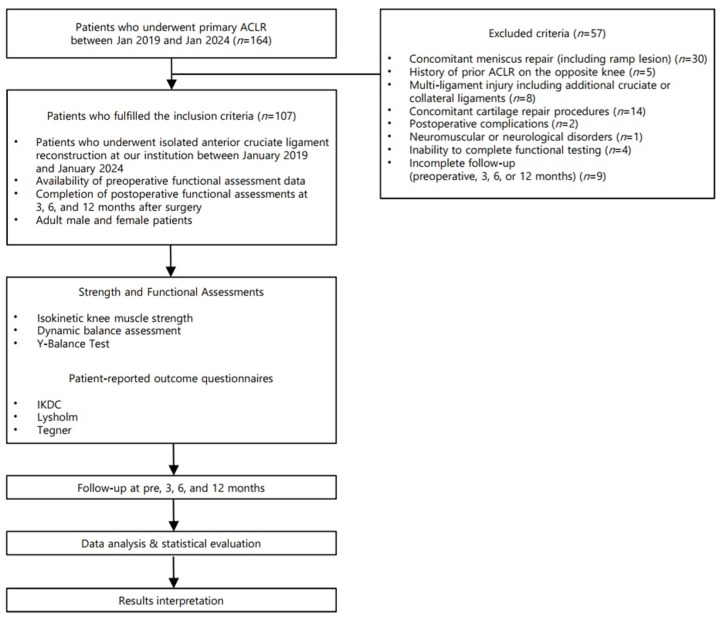
Flowchart of the study design, including patient inclusion and exclusion, strength and functional assessments, and patient-reported outcome questionnaires at each time point following anterior cruciate ligament reconstruction. ACLR, anterior cruciate ligament reconstruction; IKDC, International Knee Documentation Committee score.

After applying the exclusion criteria, 107 patients were deemed eligible and included in the final analysis. Missing data were managed using a complete-case approach. Of the 164 initially screened patients, 57 were excluded according to predefined criteria, including 9 patients with incomplete follow-up data at one or more assessment time points. No statistical imputation methods were applied. The final analysis included only patients with complete datasets across all predefined assessment time points. The process of patient inclusion and exclusion and assessment time points are summarized in Figure 1.

This retrospective study was conducted in accordance with the Declaration of Helsinki and was approved by the Institutional Review Board of Konkuk University Medical Center (KUMC 2025-11-032 on 24 November 2025). Given its retrospective design, informed consent was obtained from all participants, either directly or via prior institutional consent procedures.

### 2.2. Demographic and Clinical Data

Demographic and clinical variables were collected to comprehensively describe the baseline characteristics of the study population. The demographic data included age, sex, height, weight, and body mass index. Clinical variables included pre-injury activity level assessed using the Tegner activity scale, and the time interval from injury to surgery (days). These variables were collected to characterize both the physical profile and functional background of the participants prior to surgery (Table 1).

### 2.3. Surgical Procedure

Arthroscopic ACLR was performed with a hamstring tendon autograft. The procedure was undertaken no earlier than 3 weeks following injury, after restoration of full knee extension and knee flexion of at least 120°. After femoral and tibial tunnel creation and graft passage, femoral fixation was achieved using a cortical suspensory fixation device (XO Button; ConMed, Largo, FL, USA) in combination with a transverse fixation system (Bio-Cross Pin [RIGIDFIX]; DePuy, Raynham, MA, USA). Tibial fixation was accomplished with a bioabsorbable interference screw (Matryx; ConMed, Largo, FL, USA), with supplemental fixation using a cortical screw and washer [13].

### 2.4. Postoperative Rehabilitation

All patients followed a standardized postoperative rehabilitation protocol implemented at our institution [14]. Early rehabilitation was initiated on postoperative day 3. Patients were allowed to bear weight as tolerated immediately after surgery while wearing an ACL-supporting knee brace (Legend; DonJoy, Lewisviile, TX, USA) and to start isometric quadriceps exercise and range of motion (ROM) exercise as tolerated immediately after ACLR. In addition, ROM exercises gradually progressed with the goal of achieving 120° of knee flexion by 12 weeks postoperatively. The knee brace was completely discontinued at approximately 8 weeks after surgery. Closed kinetic chain exercises (CKC) were started at 2 weeks, and Open kinetic chain (OKC) strengthening exercises and perturbation-based neuromuscular training were initiated at 6 weeks postoperatively. After 12 weeks, progressively weighted CKC and OKC exercises without ROM limitation were allowed. At 3 months postoperatively, running was permitted, provided that strength and functional performance reached at least 70% of the contralateral limb. Return to competitive sports participation was allowed no earlier than 9 months after surgery. The overall postoperative rehabilitation protocol is summarized in detail in Table 2. Patients were instructed to follow the standardized rehabilitation protocol as outlined. Supervision was provided during scheduled outpatient visits according to institutional practice; however, adherence to home-based exercise components was not formally quantified or objectively monitored.

### 2.5. Lower Limb Strength Measurement

**Figure 2:**
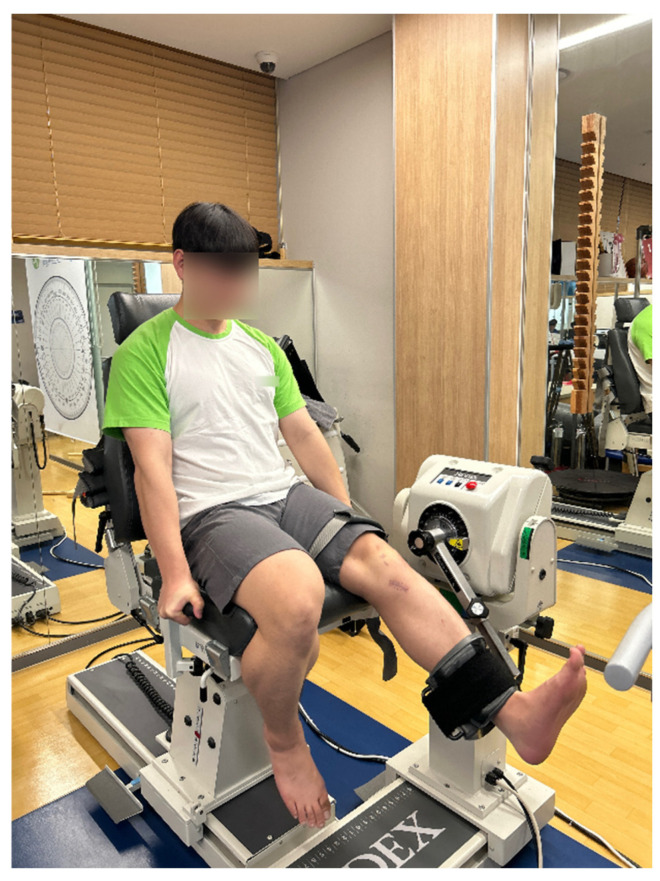
Assessment of isokinetic knee extension and flexion strength using the Biodex System 4 Pro dynamometer.

Knee isokinetic muscle strength measurements were performed using a Biodex System 4 Pro (Shirley, NY, USA) dynamometer. Subjects were seated and positioned so that the center of the lateral femoral epicondyle was aligned with the dynamometer’s axis of rotation. ROM was restricted to 0°~90° to prevent excessive flexion or extension. The knee flexion and extension torques were evaluated in isokinetic conditions with the commonly used constant angular velocities of 60°/s and 180°/s. The test included a series of 4 extending and flexing movements at the velocity of 60°/s, and 15 attempts at the velocity of 180°/s (Figure 2) [1]. The peak torque values from the trials were recorded. Strength differences between the operated and non-operated limbs were expressed as percentage deficits, calculated as [(non-operated limb peak torque − operated limb peak torque) ÷ non-operated limb peak torque] × 100.

### 2.6. Postural Stability Measurement Under Unstable Support Conditions

**Figure 3:**
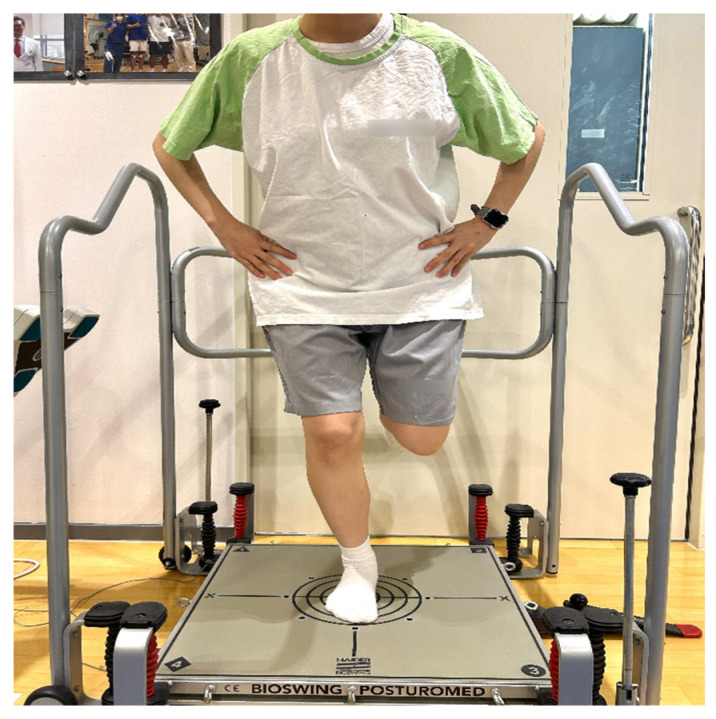
Single-leg stance assessment on the POSTUROMED platform with sway path recorded using the CMS-10 ultrasound-based motion analysis system (Zebris Medical GmbH, Isny im Allgäu, Germany).

Postural stability under unstable support conditions was evaluated using the POSTUROMED platform (Haider Bioswing, Pullenreuth, Germany) combined with a CMS-10 ultrasound-based motion analysis system and WinPosture software (Version 0.0.16, Zebris Medical GmbH, Isny im Allgäu, Germany). The POSTUROMED provides a mechanically unstable support surface that allows multi-directional oscillations, thereby requiring continuous postural stabilization to maintain balance. Participants stood barefoot at the center of the platform with their second toes aligned to the reference line. With the knee flexed to approximately 30° and eyes open, subjects performed a single-leg stance on the affected limb [15,16]. During the single-leg stance assessment, the contralateral knee was maintained in a comfortable flexed position without contacting the stance limb. The knee flexion angle was not standardized, and no goniometric measurement was performed. Once stabilized, participants released their hands and attempted to minimize sway for 10 s [17]. Sway path (in millimeters) was recorded using a CMS-10 ultrasound-based motion analysis system in combination with WinPosture software. Two active ultrasound markers fixed to the platform transmitted positional data to a three-receiver CMS-10 system at a sampling frequency of 50 Hz. The system quantified platform oscillations to calculate sway path length. Although the system internally evaluates antero-posterior and medio-lateral movements, only the overall sway path is provided and recorded for analysis. Differences between the affected and unaffected limbs were expressed as a percentage (Figure 3).

### 2.7. Dynamic Functional Performance Measurement

**Figure 4:**
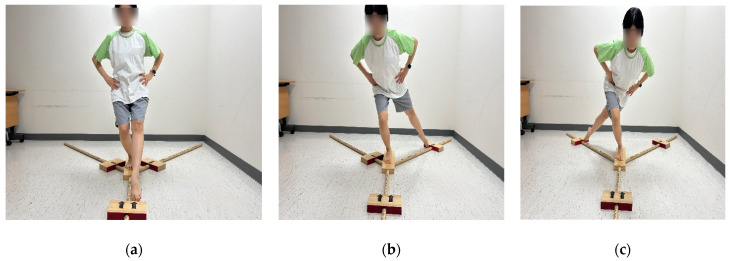
Dynamic balance assessment using the Y-Balance Test in three reach directions: (**a**) anterior, (**b**) posteromedial, and (**c**) posterolateral.

Dynamic balance was assessed using the YBT, conducted with the YBT Kit (Functional Movement Systems, Chatham, VA, USA). Each participant completed the test three times in three different directions: anterior, posteromedial, and posterolateral (Figure 4). Testing commenced with the uninvolved leg, followed by the involved leg. The greatest reach distance achieved in each direction across the three trials was recorded. These measurements were aggregated to compute a composite reach distance using the formula: [(anterior + posteromedial + posterolateral)/(3 × leg length)] × 100. The limb symmetry index (LSI) was calculated to compare the ratio between the involved and uninvolved knees [18]. The LSI was computed using the following formula: LSI(%) = (performance of the involved limb/performance of the uninvolved limb) × 100.

### 2.8. Subjective Knee Function Measurement

The patient’s subjective knee function was assessed using the Lysholm score, the International Knee Documentation Committee (IKDC) score, and the Tegner activity scale [19,20,21]. These validated questionnaires were used to evaluate symptoms, functional status, and activity level related to knee function.

### 2.9. Statistical Analysis

All statistical analyses were performed using IBM SPSS Statistics software (version 25.0; IBM Corp., Armonk, NY, USA). The normality of continuous variables was assessed using the Shapiro–Wilk test (superscript “a” in the tables denotes variables that do not satisfy normality, and superscript “b” in the tables denotes variables that satisfy normality).

For variables that did not satisfy normality assumptions, changes in outcome measures across time points (post-injury, 3, 6, and 12 months postoperatively) were analyzed using the Friedman test. Post hoc pairwise comparisons were conducted using the Wilcoxon signed-rank tests. For variables that did satisfy normality assumptions, repeated-measures analysis of variance (RM-ANOVA) was applied, followed by the Bonferroni correction for post hoc comparisons. Statistical significance was set at *p* < 0.05.

## 3. Results

### 3.1. Changes in Knee Muscle Strength

Isokinetic knee muscle strength demonstrated significant time-dependent changes following ACLR. At an angular velocity of 60°/s, for knee extension, the involved limb peak torque decreased significantly at postoperative 3 months compared with post-injury values, followed by progressive improvement at 6 and 12 months. In parallel, the uninvolved limb demonstrated a gradual increase over time, plateauing at 6 months. Consequently, the extensor deficit worsened at 3 months and then substantially decreased by 12 months, with significant between-time point differences as shown in Table 3 (*p* < 0.05). For knee flexion, the involved limb showed a small reduction at 3 months and then increased through 12 months, while the uninvolved limb also increased over time, plateauing at 6 months. The flexor deficit decreased from post-injury at 12 months, with significant differences across most time point comparisons (*p* < 0.05), except where noted in the table (Table 3).

At an angular velocity of 180°/s, significant time-dependent changes were observed in both knee extensor and flexor strength, as well as in strength deficits between the involved and uninvolved limbs. For knee extension, peak torque of the involved limb decreased slightly from post-injury values at 3 months postoperatively, followed by a significant increase at 6 months and 12 months postoperatively. In contrast, knee extensor strength of the uninvolved limb showed a gradual increase over time, plateauing at 6 months. Consequently, the extensor strength deficit peaked at 3 months postoperatively and progressively decreased at 6 months and 12 months, although a residual deficit remained at the final follow-up. For knee flexion, peak torque of the involved limb showed no significant change between post-injury assessment and 3 months postoperatively, but increased significantly at 6 months and 12 months postoperatively, while the uninvolved limb also increased over time, plateauing at 6 months. The flexor strength deficit increased at 3 months postoperatively compared with post-injury values, and subsequently decreased at 6 months and 12 months, indicating substantial recovery of flexor strength symmetry over time (Table 4).

### 3.2. Change in Postural Stability Under Unstable Support Conditions

Postural stability under unstable support conditions, assessed by sway path length during single-leg stance, demonstrated significant time-dependent changes following ACLR (Table 5). In the involved limb, sway path length significantly decreased over time, indicating progressive improvement in postural stability. Compared with the post-injury value, the sway path was significantly reduced at postoperative 3 months, 6 months, and 12 months (all *p* < 0.05). Additionally, significant reductions were observed between postoperative 3 and 6 months, as well as between 3 and 12 months. However, the difference between postoperative 6 and 12 months did not reach statistical significance (*p* = 0.163).

In the uninvolved limb, the sway path also showed a significant reduction over time when compared with the post-injury value (*p* < 0.05). However, no statistically significant differences were observed between postoperative time points (3, 6, and 12 months; all *p* > 0.05), indicating an early improvement followed by stabilization.

Significant differences in LSI were observed across most time points (*p* < 0.05), although no significant changes were found between post-injury and 3-month assessments or between post-injury and 6-month assessments. By 12 months postoperatively, LSI values approached closer to symmetry.

### 3.3. Change in Dynamic Functional Performance

YBT performance demonstrated significant time-dependent changes in LSI across all reach directions and composite scores following ACLR (Table 6).

For the anterior reach direction, LSI significantly decreased at postoperative 3 months compared with the post-injury value, followed by a gradual increase at postoperative 6 months and a significant improvement at postoperative 12 months (*p* < 0.05 for all comparisons, except post-injury vs. postoperative 6 months, *p* = 0.271).

For the posteromedial reach direction, LSI showed no significant change between post-injury assessment and postoperative 3 months (*p* = 0.167) or postoperative 6 months (*p* = 0.144). However, a significant increase was observed at postoperative 12 months compared with earlier time points (*p* < 0.05).

Similarly, posterolateral reach LSI demonstrated significant improvements over time, with no significant difference between post-injury and postoperative 6-month assessments (*p* = 0.138), but a significant increase at postoperative 12 months compared with earlier evaluations (*p* < 0.05).

The composite score LSI followed a comparable pattern, showing an initial decrease at postoperative 3 months, recovery by postoperative 6 months, and a significant increase at postoperative 12 months (*p* < 0.05 for all comparisons, except post-injury vs. postoperative 6 months, *p* = 0.088).

### 3.4. Change in Subjective Knee Function Assessments

Subjective knee function significantly improved over time following ACLR, as assessed by the Lysholm score, IKDC score, and Tegner activity scale (Table 7 and Table 8).

For the Lysholm score, no significant difference was observed between post-injury assessment and postoperative 3 months (*p* = 0.058). However, a significant improvement was observed at postoperative 6 months compared with both post-injury and 3-month assessments (all *p* < 0.05). The Lysholm score further increased at postoperative 12 months, showing significant improvement compared with all earlier time points (all *p* < 0.05).

Similarly, the IKDC score did not show a significant change between post-injury assessment and postoperative 3 months (*p* = 0.105). In contrast, significant improvements were observed at postoperative 6 months compared with both post-injury and 3-month assessments (all *p* < 0.05). The IKDC score continued to improve at postoperative 12 months, demonstrating significant differences compared with all preceding time points (all *p* < 0.05). Overall, subjective knee function scores demonstrated a gradual improvement over time, with clinically meaningful recovery becoming apparent from 6 months postoperatively rather than during the early postoperative phase (Table 7).

Changes in activity level, assessed using the Tegner activity scale. The Tegner score significantly decreased from the pre-injury level to post-injury assessment (*p* < 0.05). Postoperatively, the Tegner score gradually increased, with statistically significant differences observed between consecutive postoperative time points and relative to post-injury assessment (all *p* < 0.05). By 12 months postoperatively, the Tegner activity score had recovered to a level comparable to the pre-injury status, indicating an overall restoration of self-reported activity participation (Table 8).

### 3.5. Power of the Study

A post hoc power analysis showed that the sample size of 107 patients provided adequate statistical power (0.97) based on the comparison of the composite score LSI in dynamic functional assessment.

## 4. Discussion

The principal findings of this retrospective longitudinal cohort study were that muscle strength, postural stability, YBT performance, and subjective knee function all demonstrated significant time-dependent changes during the first year following ACLR. First, isokinetic knee extensor and flexor strength of the operated limb showed an initial decline at 3 months postoperatively, followed by progressive recovery at 6 and 12 months; however, measurable strength deficits relative to the uninvolved limb persisted at 12 months. Second, postural stability, assessed under unstable support conditions using sway path length, decreased significantly over time in both limbs. Greater reductions were observed in the operated limb, and LSIs gradually approached normalization by 12 months. Third, YBT LSIs exhibited a non-linear recovery pattern, characterized by transient deterioration or minimal improvement during the early and intermediate postoperative phases and significant improvements primarily at 12 months postoperatively. Finally, subjective knee function scores, including Lysholm and IKDC scores, did not differ significantly between post-injury assessment and 3 months postoperatively but improved markedly from 6 months onward, whereas activity level assessed by the Tegner scale gradually recovered and reached values comparable to pre-injury levels by 12 months.

In the present study, knee extensor strength recovery after ACLR was incomplete during the early and mid-postoperative phases. Extensor strength asymmetry transiently worsened at 3 months postoperatively and showed progressive improvement thereafter; however, near-symmetrical recovery was achieved only at the 12-month follow-up. These findings are consistent with previous studies reporting that quadricep strength recovery after ACLR requires a prolonged rehabilitation period and frequently remains below normative or recommended thresholds during the first 6–9 months after surgery [22,23,24]. The prior literature has demonstrated that quadricep strength deficits are more pronounced and persistent than hamstring deficits following hamstring autograft ACLR, with reported recovery rates for the quadriceps of approximately 70% at 6 months and 80% at 12 months [25]. In line with these observations, the present study demonstrated a comparable recovery trajectory, with quadricep strength reaching approximately 76% at 6 months and approaching 87% at 12 months relative to the contralateral limb. LSI values are commonly interpreted using a ≥90% threshold as a benchmark for RTS decision-making. Previous studies have shown that failing to achieve ≥90% symmetry before RTS is associated with a significantly increased risk of re-injury following ACLR [26,27]. Therefore, LSI values below this threshold may indicate residual functional asymmetry with potential clinical implications. Persistent quadricep strength deficits at 12 months may remain clinically meaningful, as residual asymmetry has been associated with altered movement mechanics and increased re-injury risk [28,29]. These findings suggest that temporal recovery does not necessarily reflect complete neuromuscular restoration and support the need for continued strength-focused rehabilitation beyond the first postoperative year.

The delayed recovery of knee extensor strength observed in this study may be explained by multiple interacting mechanisms. Early postoperative quadricep weakness has been attributed to arthrogenic muscle inhibition (AMI) induced by pain, joint effusion, inflammation, and altered afferent input following ACL injury and reconstruction [30,31,32]. Experimental and clinical studies have shown that quadricep activation failure can persist even after pain and swelling subside, contributing to prolonged strength deficits despite ongoing rehabilitation [31,32]. In addition, reduced mechanical loading and decreased physical activity during the early postoperative phase may further exacerbate muscle atrophy and delay neuromuscular recovery [23,33].

Importantly, previous longitudinal studies have suggested that improvements in patient-reported knee function may precede measurable gains in quadriceps strength and strength symmetry [34]. This dissociation underscores the limitation of relying solely on subjective outcomes or time-based criteria to infer neuromuscular recovery. Collectively, the present findings indicate that meaningful recovery of knee extensor strength after ACLR requires at least 6 months, with continued improvements extending up to 12 months postoperatively. These results support the need for prolonged, strength-focused rehabilitation strategies and caution against premature assumptions of functional recovery based on early postoperative patient-reported outcome improvements.

In contrast to knee extensor strength, recovery of knee flexor strength following ACLR using a hamstring tendon autograft appears to follow a different and generally more favorable recovery pattern. Previous isokinetic studies have consistently shown that postoperative deficits in knee flexor strength are smaller than those observed in knee extensors, which has supported the widespread use of hamstring tendons as grafts to minimize donor-site morbidity [35].

Nevertheless, transient hamstring weakness is commonly observed during the early postoperative period. Several studies have reported reduced hamstring peak torque and decreased hamstring-to-quadriceps (H/Q) ratios at approximately 3–6 months after ACLR, particularly when the semitendinosus tendon is harvested [36,37,38]. These deficits are thought to reflect incomplete recovery of the harvested semitendinosus and gracilis muscles, as well as alterations in neuromuscular control rather than true improvements in absolute flexion strength [36,37].

Longitudinal studies suggest that hamstring strength deficits progressively decrease over time. For example, flexion strength deficits at 60°/s have been reported to decline from approximately 15–20% at 6 months to less than 5% by 12 months postoperatively [38]. However, early recovery remains limited, with LSI for knee flexor strength reported to be as low as 54–70% during the first 8–12 weeks following ACLR with hamstring autografts [39].

The persistence of early flexor strength deficits may be further influenced by cautious rehabilitation strategies. Open kinetic chain hamstring strengthening exercises are often delayed until 6–8 weeks postoperatively to protect the graft harvest site, potentially contributing to delayed restoration of flexion strength, particularly at deeper knee flexion angles [40,41,42].

Taken together, these findings indicate that although knee flexor strength generally recovers more rapidly and to a greater extent than knee extensor strength after ACLR, clinically relevant deficits persist during the early postoperative period. Targeted and progressive rehabilitation strategies remain essential to optimize hamstring recovery and restore balanced knee muscle function.

In the present study, postural stability, as assessed by sway path parameters, demonstrated a gradual improvement over time following ACLR; however, recovery was incomplete during the early and mid-postoperative periods. Specifically, the sway path length of the involved limb decreased progressively from post-injury assessment to 12 months postoperatively, indicating an overall improvement in postural control capacity. Nevertheless, significant limb asymmetry persisted during the 6-month postoperative interval, as reflected by unstable and highly variable LSI values, suggesting incomplete restoration of symmetrical balance control during this phase.

These findings are consistent with previous longitudinal studies indicating that postural stability after ACLR improves gradually over time and may remain incomplete during the first postoperative year. Brophy et al. reported progressive improvements in postural stability up to 12 months postoperatively, with additional gains observed between 12 and 24 months, and noted plane-specific differences in recovery patterns [43]. These observations suggest that balance restoration after ACLR is time-dependent and may not follow a uniform recovery pattern during the early and mid-rehabilitation phases. Importantly, although different balance assessment tools were used, the temporal pattern of delayed stabilization aligns closely with the present results [44]. The incomplete recovery of balance during the 3–6-month postoperative period may be explained by persistent neuromuscular and sensorimotor deficits following ACL injury and reconstruction. Several studies have shown that disruption of ACL mechanoreceptors leads to altered afferent input to the central nervous system, resulting in impaired postural regulation even after surgical stabilization [45,46,47]. Kielė et al. reported that dynamic balance during single-leg hop tasks may paradoxically worsen in the reconstructed limb during early rehabilitation, despite improvements in dynamic balance, suggesting that higher-demand dynamic tasks expose residual neuromuscular deficits that are not apparent during simpler postural tasks [45]. Another important consideration is the dissociation between balance recovery and muscular strength restoration. Prior investigations have demonstrated that postural stability depends not only on quadricep strength but also on coordinated activation of hip, ankle, and trunk musculature [47,48]. As quadricep weakness and altered movement strategies persist during the early postoperative period, patients may adopt compensatory control strategies that reduce overall sway magnitude while failing to restore true inter-limb symmetry, thereby contributing to the observed LSI instability. Taken together, the present findings suggest that although postural stability improves progressively after ACLR, balance recovery remains incomplete between 3 and 6 months postoperatively. This interpretation is supported by recent evidence indicating that monopodalic assessments may fail to detect persistent postural control alterations, whereas bipodalic dynamic conditions appear to be more sensitive in identifying residual deficits following ACLR [49]. These findings highlight the importance of considering task-specific assessment sensitivity when interpreting postural stability recovery after ACLR.

In the present study, YBT performance showed a transient deterioration at 3 months postoperatively, followed by progressive improvement at 6 and 12 months after ACLR. Anterior reach, posterolateral reach, and composite scores decreased at 3 months compared with post-injury assessment, whereas posteromedial reach did not show a significant early decline. By 12 months, all reach directions demonstrated significant improvement, indicating a time-dependent but direction-specific recovery pattern.

The early reduction in anterior reach performance observed at 3 months is likely related to postoperative quadriceps weakness. Previous studies have consistently shown that anterior reach distance is strongly associated with knee extensor strength and sagittal-plane knee control following ACLR [11,50]. In particular, several investigations reported that reductions in quadricep strength during the early postoperative period are accompanied by decreased anterior reach symmetry, even when other functional measures appear preserved [51]. Therefore, the deterioration in anterior reach observed in the present study at 3 months is consistent with the known time course of quadricep strength suppression after ACLR.

In contrast, posteromedial reach did not exhibit a significant decline at 3 months and recovered to post-injury levels by 6 months, with further improvement at 12 months. This finding may be explained by the biomechanical characteristics of the posteromedial direction, which places greater demands on frontal-plane pelvic stability and hip abductor function rather than isolated knee extensor strength [52,53]. Previous studies have demonstrated that posteromedial reach performance is closely related to gluteus medius strength and proximal neuromuscular control [53,54]. Given that early rehabilitation protocols after ACLR commonly emphasize hip and core strengthening, preservation of posteromedial reach during the early postoperative period may reflect compensatory proximal adaptations rather than full recovery of knee function.

Notably, the previous literature has reported heterogeneous findings regarding early YBT recovery after ACLR. Some studies observed improvements in Y-Balance performance within the first 3 months despite persistent strength deficits, suggesting early neuromuscular adaptation [51]. Conversely, other investigations reported that Y-Balance symmetry does not significantly improve between 4 and 6 months postoperatively, indicating delayed restoration of dynamic balance [55]. The present findings align more closely with the latter perspective, supporting the notion that dynamic balance recovery following ACLR is not linear and varies according to reach direction and postoperative time point. In support of this interpretation, Barzyk et al. reported that although composite YBT scores improved over time, individual reach directions did not demonstrate consistent changes, highlighting direction-specific variability and differences in measurement sensitivity. Moreover, the predominantly weak associations between YBT scores and strength or functional performance measures suggest that the YBT primarily reflects dynamic postural control rather than comprehensive functional recovery [11]. These findings underscore the importance of considering task- and metric-specific characteristics when interpreting YBT outcomes after ACLR. Subjective knee function improved over time after ACLR, with substantial recovery occurring mainly after 6 months and continuing through 12 months postoperatively.

These findings are consistent with previous studies reporting that patient-reported outcomes show limited improvement during the early postoperative period following ACLR. Several longitudinal investigations have demonstrated that Lysholm and IKDC scores often remain unchanged or show only minimal improvement within the first 3–4 months after surgery, reflecting persistent pain, perceived instability, and activity restrictions during this phase [56,57,58]. Agarwalla et al. reported that maximal improvements in subjective knee function typically occur between 6 and 12 months postoperatively [59], while other studies have similarly described a delayed recovery trajectory in which clinically meaningful improvements emerge only after the mid-postoperative period [60].

The delayed improvement in subjective knee function observed in the present study may be partly attributable to incomplete recovery of knee extensor strength and dynamic balance during the early and mid-postoperative stages. Previous research has consistently demonstrated strong associations between quadricep strength deficits, reduced functional performance, and lower patient-reported outcome scores following ACLR [56]. In particular, Menzer et al. identified objective deficits in muscle strength and functional performance during the first postoperative year as significant predictors of subjective knee function [57].

During the 6–12-month postoperative period, concurrent improvements in muscle strength, balance performance, and overall functional capacity may have contributed to the observed enhancement in subjective knee function scores. Several studies have reported continued improvements in patient-reported outcomes beyond 6 months postoperatively, emphasizing the close relationship between neuromuscular recovery, movement control, and perceived knee function [59,60,61]. Moreover, patients’ confidence and perception regarding RTS appear to develop progressively over time, further supporting the clinical importance of a 1-year follow-up when evaluating functional recovery after ACLR [58]. The non-synchronous recovery patterns observed across muscle strength, postural stability, YBT performance, and patient-reported outcomes likely reflect distinct yet interrelated physiological mechanisms. Quadricep strength recovery is primarily constrained by arthrogenic muscle inhibition and altered mechanical loading in the early postoperative phase, whereas dynamic balance and YBT performance rely more heavily on sensorimotor integration and multi-joint neuromuscular coordination. Consequently, improvements across functional domains may not occur concurrently. Furthermore, patient-reported recovery may reflect reductions in pain and improved confidence rather than complete neuromuscular symmetry, contributing to temporal dissociation among outcome measures.

### Limitations

Several limitations of this study should be acknowledged. First, the retrospective study design limits control over potential confounding factors and precludes causal inference. Second, the study cohort exhibited an imbalance in sex distribution, and sex-specific analyses were not performed. Therefore, potential sex-related differences in recovery patterns could not be evaluated, which may limit the generalizability of the findings. Third, although follow-up assessments were conducted up to 12 months postoperatively, a longer follow-up period would be necessary to fully characterize long-term recovery patterns. Fourth, pre-injury baseline data for muscle strength, balance, functional performance, and clinical outcome measures were not available. Post-injury assessments were performed at an average of approximately six weeks after injury; therefore, the true pre-injury functional status could not be determined. Consequently, it remains difficult to identify when or whether postoperative recovery reaches the actual pre-injury level. Fifth, inclusion of patients who underwent concomitant minimal partial meniscetomy may have confounded the study outcomes. However, we believed this did not alter the postoperative rehabilitation protocol and that its effect would be minimal. Sixth, limb dominance was not recorded in the retrospective dataset. Given that limb dominance may influence recovery thresholds and the interpretation of limb symmetry indices following ACLR, the absence of this information should be considered when interpreting the present findings. Finally, rehabilitation-related factors, including exercise adherence and progression, were not formally quantified or controlled, which may have contributed to inter-individual variability in the observed outcomes.

## 5. Conclusions

This study observed serial changes in muscle strength and functional ability during the first year following ACLR. Recovery appears to remain incomplete during the early to mid-postoperative period, with both muscle strength and functional performance showing substantial impairments between 3 and 6 months after surgery. Although gradual improvements were observed thereafter, they continued up to 12 months, and complete restoration of knee extensor strength symmetry was not achieved at this time point. Functional ability, including dynamic balance and lower-limb performance, improved at a later stage compared to strength recovery, and patient-reported knee function followed a similar trajectory. Collectively, these findings suggest that functional restoration after ACLR may require a prolonged rehabilitation process beyond the early postoperative phase and highlight the importance of serial, integrated assessments of muscle strength and functional ability when interpreting postoperative recovery.

## Figures and Tables

**Table 1:** Demographic and clinical characteristics of the study participants.

*n* = 107	Mean ± SD
Age at surgery (year)	29.98 ± 11.91
Gender (M/F)	80/27
Pre-injury Tegner activity scale	5.8± 1.8
Time from injury to surgery (day)	53.63 ± 36.54
Height (cm)	172.48 ± 9.33
Weight (kg)	75.13 ± 14.11
BMI (kg/m^2^)	25.17 ± 3.55

SD, standard deviation; M/F, male/female; BMI, body mass index.

**Table 2:** Rehabilitation program after anterior cruciate ligament reconstruction.

	Time						
Rehabilitation	0–3 wks	4–6 wks	7–9 wks	10–12 wks	3 mo	6 mo	9 mo
Range of motion							
0–90° (progressed by 30° per week)	O						
0–120°		O	O	O	O	O	O
Weight bearing	PWB with brace	FWB with brace	FWB				
Strengthening							
Quadriceps setting	O	O					
Ankle pump	O						
Straight leg raising 4 way	O	O					
Knee extension with resistance band		O					
Knee flexion with resistance band			O	O			
Leg extension machine			O	O	O	O	O
Leg curl machine					O	O	O
Calf raise	O						
Mini squat (0–45°)	O						
Squat		O	O				
Lunge			O	O			
Multi-directional single squats				O	O	O	O
Deadlifts (hamstring)					O	O	O
Nordic hamstring curl						O	O
Proprioception training							
Single-leg balance		O	O				
Unstable single-leg balance			O	O	O	O	O
Perturbation training				O	O	O	O
Functional exercises							
Normal walking		O					
Stair		O	O				
Bilateral landing				O	O	O	O
Light running					O	O	O
Plyometric					O	O	O
Agility						O	O
Return to sports							O

PWB, partial weight bearing; FWB, full weight bearing; wks, weeks; mo, months.

**Table 3:** Changes in peak knee extensor and flexor strength and strength deficits at 60°/s across time points.

Strength	Extension			Flexion		
(60°/s)	Involved (Nm) ^b^	Uninvolved (Nm) ^a^	Deficit (%) ^a^	Involved (Nm) ^b^	Uninvolved (Nm) ^a^	Deficit (%) ^a^
Post-injury	120.70 ± 50.81 ^2,3,4^	178.15 ± 48.87 ^2,3,4^	30.85 ± 23.00 ^2,3,4^	67.82 ± 29.76 ^3,4^	85.10 ± 26.89 ^2,3,4^	21.13 ± 23.55 ^3,4^
PO 3 month	114.19 ± 44.40 ^1,3,4^	186.45 ± 51.99 ^1,3,4^	37.97 ± 18.18 ^1,3,4^	65.25 ± 23.92 ^3,4^	87.18 ± 27.92 ^1,3,4^	24.71 ± 18.12 ^3,4^
PO 6 month	147.08 ± 53.88 ^1,2,4^	193.26 ± 52.46 ^1,2^	23.51 ± 18.33 ^1,2,4^	81.49 ± 29.07 ^1,2,4^	92.94 ± 28.35 ^1,2^	11.71 ± 18.16 ^1,2,4^
PO 12 month	169.43 ± 59.86 ^1,2,3^	195.86 ± 56.56 ^1,2^	13.66 ± 17.42 ^1,2,3^	88.43± 31.70 ^1,2,3^	97.31 ± 34.61 ^1,2^	7.62 ± 13.98 ^1,2,3^

Superscript a: the Friedman test was used for analysis, and post hoc pairwise comparisons were conducted using the Wilcoxon signed-rank tests. Superscript b: repeated-measures analysis of variance was applied for analysis, followed by the Bonferroni correction for post hoc comparisons. Superscript numbers (^1–4^) indicate time points at which statistically significant differences were observed (*p* < 0.05): ^1^ post-injury, ^2^ postoperative 3 months, ^3^ postoperative 6 months, and ^4^ postoperative 12 months; PO, postoperative.

**Table 4:** Changes in peak knee extensor and flexor strength and strength deficits, at 180°/s across time points.

Strength	Extension			Flexion		
180°/s	Involved (Nm) ^b^	Uninvolved (Nm) ^a^	Deficit (%) ^a^	Involved (Nm) ^a^	Uninvolved (Nm) ^a^	Deficit (%) ^a^
Post-injury	88.66 ± 32.86 ^3,4^	117.58 ± 35.73 ^2,3,4^	23.54 ± 19.95 ^2,4^	50.88 ± 18.96 ^3,4^	60.80 ± 19.20 ^2,3,4^	15.08 ± 19.93 ^2,4^
PO 3 months	84.07 ± 30.85 ^3,4^	125.55 ± 37.12 ^1,3,4^	32.42 ± 16.97 ^1,3,4^	50.70 ± 17.80 ^3,4^	63.64 ± 19.41 ^1,3,4^	19.68 ± 16.83 ^1,3,4^
PO 6 months	100.07 ± 37.84 ^1,2,4^	128.38 ± 36.54 ^1,2^	20.37 ± 17.10 ^2,4^	59.21 ± 18.97 ^1,2,4^	66.39 ± 19.34 ^1,2^	8.75 ± 15.67 ^2,4^
PO 12 months	118.79 ± 39.48 ^1,2,3^	131.86 ± 39.05 ^1,2^	11.66 ± 16.12 ^1,2,3^	66.12 ± 20.98 ^1,2,3^	70.23 ± 22.07 ^1,2^	4.68 ± 14.86 ^1,2,3^

Superscript a: the Friedman test was used for analysis, and post hoc pairwise comparisons were conducted using the Wilcoxon signed-rank tests. Superscript b: repeated-measures analysis of variance was applied for analysis, followed by the Bonferroni correction for post hoc comparisons. Superscript numbers (^1–4^) indicate time points at which statistically significant differences were observed (*p* < 0.05): ^1^ post-injury, ^2^ postoperative 3 months, ^3^ postoperative 6 months, and ^4^ postoperative 12 months; PO, postoperative.

**Table 5:** Change in postural stability across time points.

Postural Stability			
	Involved (mm) ^a^	Uninvolved (mm) ^a^	LSI (%) ^a^
Post-injury	163.95 ± 144.52 ^2,3,4^	131.07 ± 127.08 ^2,3,4^	162.96 ± 133.38 ^4^
PO 3 months	141.30 ± 178.67 ^1,3,4^	101.46 ± 127.10 ^1^	187.19 ± 171.59 ^3,4^
PO 6 months	95.55 ± 120.13 ^1,2^	87.35 ± 110.55 ^1^	166.68 ± 231.41 ^2,4^
PO 12 months	74.03 ± 71.31 ^1,2^	84.23 ± 90.53 ^1^	111.72 ± 113.00 ^1,2,3^

Superscript a: the Friedman test was used for analysis, and post hoc pairwise comparisons were conducted using the Wilcoxon signed-rank tests. Superscript numbers (^1–4^) indicate time points at which statistically significant differences were observed (*p* < 0.05): ^1^ post-injury, ^2^ postoperative 3 months, ^3^ postoperative 6 months, and ^4^ postoperative 12 months; LSI, limb symmetry index; PO, postoperative.

**Table 6:** Change in YBT performance across time points.

Dynamic Functional Assessment				
	Anterior Reach LSI (%) ^a^	PM Reach LSI (%) ^a^	PL Reach LSI (%) ^a^	Composite Score LSI (%) ^a^
Post-injury	92.66 ± 6.89 ^2,4^	96.23 ± 7.11 ^4^	95.86 ± 7.25 ^2,4^	95.19 ± 5.38 ^2,4^
PO 3 months	88.76 ± 8.88 ^1,3,4^	95.15 ± 8.01 ^3,4^	93.49 ± 5.51 ^1,3,4^	93.05 ± 5.67 ^1,3,4^
PO 6 months	93.24 ± 7.88 ^2,4^	97.59 ± 7.16 ^2,4^	96.79 ± 8.85 ^2,4^	96.44 ± 6.90 ^2,4^
PO 12 months	97.53 ± 7.98 ^1,2,3^	99.33 ± 5.40 ^1,2,3^	99.25 ± 6.94 ^1,2,3^	98.83 ± 4.99 ^1,2,3^

Superscript a: the Friedman test was used for analysis, and post hoc pairwise comparisons were conducted using the Wilcoxon signed-rank tests. Superscript numbers (^1–4^) indicate time points at which statistically significant differences were observed (*p* < 0.05): ^1^ post-injury, ^2^ postoperative 3 months, ^3^ postoperative 6 months, and ^4^ postoperative 12 months. PM, posteromedial; PL, posterolateral; LSI, limb symmetry index; PO, postoperative.

**Table 7:** Change in subjective knee function score (Lysholm and IKDC) across time points.

	Subjective Knee Function Score	
	Lysholm ^a^	IKDC ^a^
Post-injury	68.54 ± 15.54 ^3,4^	63.64 ± 15.46 ^3,4^
PO 3 months	73.51 ± 14.00 ^3,4^	64.87 ± 16.41 ^3,4^
PO 6 months	86.42 ± 10.72 ^1,2,4^	77.06 ± 14.83 ^1,2,4^
PO 12 months	90.69 ± 11.17 ^1,2,3^	85.58 ± 16.06 ^1,2,3^

Superscript a: the Friedman test was used for analysis, and post hoc pairwise comparisons were conducted using the Wilcoxon signed-rank tests. Superscript numbers (^1–4^) indicate time points at which statistically significant differences were observed (*p* < 0.05): ^1^ post-injury, ^2^ postoperative 3 months, ^3^ postoperative 6 months, and ^4^ postoperative 12 months. IKDC, International Knee Documentation Committee score; PO, postoperative.

**Table 8:** Change in subjective knee function score (Tegner) across time points.

	Subjective Knee Function Score
	Tegner Activity Score ^a^
Pre-injury	5.8 ± 1.8 ^2,3,4^
Post-injury	2.9 ± 0.5 ^1,3,4,5^
PO 3 months	3.9 ± 1.0 ^1,2,4,5^
PO 6 months	5.3 ±1.3 ^1,2,3,5^
PO 12 months	6.3 ± 1.9 ^2,3,4^

Superscript a: the Friedman test was used for analysis, and post hoc pairwise comparisons were conducted using the Wilcoxon signed-rank tests. Superscript numbers (^1–5^) indicate time points at which statistically significant differences were observed (*p* < 0.05): ^1^ pre-injury, ^2^ post-injury, ^3^ postoperative 3 months, ^4^ postoperative 6 months, and ^5^ postoperative 12 months. PO, postoperative.

## Data Availability

The datasets analyzed in this study are not publicly available but are available from the corresponding author on appropriate request.
